# Non-verbal speech cues as objective measures for negative symptoms in patients with schizophrenia

**DOI:** 10.1371/journal.pone.0214314

**Published:** 2019-04-09

**Authors:** Yasir Tahir, Zixu Yang, Debsubhra Chakraborty, Nadia Thalmann, Daniel Thalmann, Yogeswary Maniam, Nur Amirah binte Abdul Rashid, Bhing-Leet Tan, Jimmy Lee Chee Keong, Justin Dauwels

**Affiliations:** 1 Institute for Media Innovation, Nanyang Technological University, Singapore, Singapore; 2 Institute of Mental Health, Singapore, Singapore; 3 Lee Kong Chian School of Medicine, Nanyang Technological University, Singapore, Singapore; 4 Singapore Institute of Technology, Singapore, Singapore; 5 School of Electrical and Electronics Engineering, Nanyang Technological University, Singapore, Singapore; Department of Psychiatry and Neuropsychology, Maastricht University Medical Center, NETHERLANDS

## Abstract

Negative symptoms in schizophrenia are associated with significant burden and possess little to no robust treatments in clinical practice today. One key obstacle impeding the development of better treatment methods is the lack of an objective measure. Since negative symptoms almost always adversely affect speech production in patients, speech dysfunction have been considered as a viable objective measure. However, researchers have mostly focused on the verbal aspects of speech, with scant attention to the non-verbal cues in speech. In this paper, we have explored non-verbal speech cues as objective measures of negative symptoms of schizophrenia. We collected an interview corpus of 54 subjects with schizophrenia and 26 healthy controls. In order to validate the non-verbal speech cues, we computed the correlation between these cues and the NSA-16 ratings assigned by expert clinicians. Significant correlations were obtained between these non-verbal speech cues and certain NSA indicators. For instance, the correlation between Turn Duration and Restricted Speech is -0.5, Response time and NSA Communication is 0.4, therefore indicating that poor communication is reflected in the objective measures, thus validating our claims. Moreover, certain NSA indices can be classified into observable and non-observable classes from the non-verbal speech cues by means of supervised classification methods. In particular the accuracy for Restricted speech quantity and Prolonged response time are 80% and 70% respectively. We were also able to classify healthy and patients using non-verbal speech features with 81.3% accuracy.

## 1 Introduction

Schizophrenia is a chronic and disabling mental disorder with heterogeneous presentations. They are characterized broadly by positive (hallucinations and delusions), negative (avolition, anhedonia, asociality, blunted affect and alogia) and cognitive (deficits in attention, memory and executive functioning) symptoms [1–4]. Unlike positive symptoms which are easily identified and effectively treated with pharmacological treatments, negative symptoms tend to be neglected with ineffective treatments [5, 6]. Moreover, negative symptoms have been consistently reported to contribute to poor function and quality of life in patients with schizophrenia [7–9] and highlighted as a significant unmet needs in a large percentage of patients [4].

Negative symptoms are assessed on clinical assessment scales (see [10] for a review) via interviews which relies on interviewer’s subjective judgement and normally takes about 15-30 minutes. The implementation of routine negative symptoms assessment in clinical practice is hampered by both training of interviewers and the time. Therefore there is a need to develop a convenient objective measurement of negative symptoms for detection of impairments and monitoring of treatment effectiveness to be used in fast-paced clinical setting.

In recent years, the advancements in speech processing research such as INTERSPEECH competitions [11, 12] and the Audio-Visual Emotion Challenge (AVEC) [13, 14] have paved the way for researchers to investigate the assessment or diagnosis of mental disorders utilizing speech analysis. Non-verbal speech or voice based cues such as prosodic features, formant features, source features and spectral analysis features were extracted from speech and machine learning models would be applied on these features to predict the existence or the severity of various mental disorders such as depression, PTSD, and autism [15–17]. The speech features could be used along with traditional clinical ratings to improve the detection of the mental illness [18].

Previous non-automated attempts to utilize the different aspects of speech and language as differentiators between individuals with schizophrenia and healthy individuals have had limited success. Although there existed some distinction in verbal fluency tasks between patients and healthy controls [19], other studies involving semantic boundary [20] or metaphor interpretation [21] reported no significant differences between the two groups. However, automated efforts based on speech deficiencies to distinguish patients and healthy controls have had greater success with the recent advancements in computer science and signal processing techniques. The ability of individuals with schizophrenia to express emotions was compared to that of a healthy group [22]. Subtle differences in communication discourses were detected among patients, their first-degree relatives and healthy controls employing Latent Semantic Analysis (LSA) in [23]. LSA was again utilized to identify lack of semantic and phonological fluency, disconnected speech, and thought disorder in [24], and LSA and machine learning were used to analyze free-speech and predict the onset of psychosis respectively of high-risk youths in [25].

However, all the above methods are based on semantic analysis and natural language processing. Restricted non-verbal cues display of patients suffering from negative symptom schizophrenia [26] have not been explored much. In [27] computerized measures of flat affect, alogia and anhedonia were examined in their relationships to clinically-rated negative symptoms and social functioning for 14 patients with flat affect, 46 patients without flat affect and 19 healthy controls. The results suggest that speech rate measure significantly discriminated patients with flat affect from controls. The computer measures of alogia and negative emotions significantly discriminated flat affect and non-flat affect patients. In [28] a system was designed to assess the speech for patient’s negative symptoms. Results show a relationship between dyadic interaction and flat affect, pause production and alogia, and rate and asociality. In [29] acoustic analysis of speech in response of visual stimuli was conducted for 48 stable outpatients for schizophrenia and mood disorders. The results show that computerized acoustic analysis appears to be a promising method for understanding the pathological manifestation of these disorders. In [30] the correlation between the variability of tongue front and back movement, and formant frequencies to the negative symptoms was studied for 25 first episode psychosis patients. In [31] interviews of 20 subjects with flat affect, 26 with non-flat affect, and 20 healthy controls were analysed to determine the motor expressive deficiency in schizophrenic patients. In [18] clinical ratings of flat affect and alogia were compared to the patient’s speech prosody and productivity. The results suggest that acoustic analysis can provide objective measures that may help in clinical assessment. In [32] a semi-automatic method was employed to quantify the degree of expressive prosody deficits in schizophrenia for 45 patients and 35 healthy controls. The results suggest that using non-verbal speech analysis the researchers were capable of classifying patient and controls with 93.8% accuracy. Non-verbal speech cues such as voice tone, volume, and interjections play a crucial role in human interaction and communication [33], and the display of such signals in patients can be used for both distinguishing them with healthy controls and developing specific and objective treatments. In existing work speech analysis has mostly been used to determine the presence and/or the severity of symptoms.

In this study we built upon our earlier work [34] and attempted to explore the utility of non-verbal speech cues of determine the severity of negative symptoms in schizophrenia. Specifically, we study the correlations between subjective ratings of negative symptoms on a clinical scale during interviews and the objective non-verbal speech features extracted from audio recording of the same interview.

## 2 Methods

### 2.1 Subjects

Fifty-four outpatients diagnosed with schizophrenia from the Institute of Mental Health, Singapore and twenty-six healthy individuals from general population were recruited in this study. The inclusion criteria of the study included diagnosis of schizophrenia for patient group or no mental illness for control group, aged 16—65, English speaking and fit to provide informed consent. The capacity of consent was assessed by asking participants to describe the purpose and procedures of the study to interviewers. All the participants finally selected for the study were consenting adults, above 18 years of age. The exclusion criteria included history of strokes, traumatic brain injuries and neurological disorders such as epilepsy. The Structured Clinical Interview for DSM-IV (SCID) was conducted for all participants to ascertain the diagnoses of schizophrenia for patients and no mental illness for healthy individuals by trained research psychologists. Ethics approval for the study was provided by the National Healthcare Group Domain Specific Review Board. Written informed consent was obtained from all participants. The sample characteristics were presented in Table 1.

**Table 1:** Sample characteristics.

	Schizophrenia sample	Healthy Control Sample	*p*-values
Gender (male:female)	25:29	12:14	0.990
Age (years)	31.06 ± 7.52	29.58 ± 8.09	0.424
Total years of education	13.67 ± 2.76	13.53 ± 2.23	0.825
Duration of illness	9.06 ± 7.36	-	
Age of illness onset	22.56 ± 5.30	-	
Medication (%)			
Antipsychotics	94.44		
Typical antipsychotics	7.41	-	
Atypical antipsychotics	81.48	-	
Anticholinergics	25.93	-	
Antidepressants	31.48	-	
Mood Stabilizers	25.93	-	
Benzodiazepine	14.81	-	
CPZ equivalence (mg/day)	412.19 ± 352.01	-	
BPRS Total Score	32.81 ± 8.86	19.81 ± 1.86	< 0.001
NSA Total Score	41.28 ± 9.39	26.77 ± 3.77	< 0.001
NSA—Communication Domain Score	7.96 ± 3.38	4.46 ± 0.71	< 0.001
NSA—Emotion Affect Domain Score	8.65 ± 1.99	6.04 ± 1.73	< 0.001
NSA—Social Involvement Domain Score	9.02 ± 2.67	7.04 ± 2.01	< 0.001
NSA—Motivation Domain Score	11.85 ± 2.68	6.92 ± 1.94	< 0.001
NSA—Retardation Domain Score	3.80 ± 1.81	2.31 ± 0.47	< 0.001

CPZ = Chloropromazine;

BPRS = The Brief Psychiatric Rating Scale;

NSA = Negative Symptom Assessment

### 2.2 Clinically-rated symptom measures

The 16-item Negative Symptom Assessment (NSA-16) [35] is a reliable and validated scale used to measure the severity of negative symptoms through semi-structured interview. It contains 16 items, each rated on a 6-point Likert scale where higher ratings indicate more severe impairments. In addition to the individual item scores, the scale also provides a global negative symptom rating (based on the overall clinical impression of negative symptoms in the patient), a total score (sum of the scores on the 16 items), and five negative symptoms domains scores including Communication (sum of the scores of item 1-4), Affect/Emotion (sum of the scores of item 5-7), Social Involvement (sum of the scores of item 8-10), Motivation (sum of the scores of item 11-14), and Retardation (sum of the scores of item 15 and 16). The NSA-16 demonstrated high internal consistency (Cronbach’s alpha = 0.92) and inter-rater reliability (Kappa value = 0.89) [36]. NSA was rated by trained research psychologists and inter-rater reliability was 0.92. The meanings of NSA items and domains are shown in Table 2.

**Table 2:** NSA items and their explanations.

Label	Criteria	Explanation
NSA 1	Prolonged time to respond	After asking the subject a question, he/she pauses for inappropriately long periods before answering
NSA 2	Restricted speech quantity	Ratings on this item suggest that the subject gives brief answers to questions and/or provides elaborating details only after the interviewer prods him
NSA 3	Impoverished speech content	The subject may talk a lot or a little but the information conveyed is very limited
NSA 4	Inarticulate speech	The subject’s speech cannot be understood because enunciation is poor
NSA 5	Emotion: Reduced range	Emotion is the feeling content of a person’s inner life. This item assesses the range of emotion experienced by the subject during the last week (or other specified time period)
NSA 6	Affect: Reduced modulation of intensity	This item assesses the subject’s modulations of intensity of affect shown during the interview while discussing matters that would be expected to elicit significantly different affective intensities in a normal person
NSA 7	Affect: Reduced display on demand	This items assesses the subject’s ability to display a range of affect as expressed by changes in his/her facial expression and gestures when asked by the interviewer to show how his/her face appears when he/she feels happy, sad, proud, scared, surprised, and angry
NSA 8	Reduced social drive	This item assesses how much the subject desires to initiate social interactions. Desire may be measured in part by the number of actual or attempted social contacts with others
NSA 9	Poor rapport with interviewer	This item assesses the interviewer’s subjective sense that he/she and the subject are actively engaged in communication with one another
NSA 10	Interest in emotional and physical intimacy	This item assesses how much the subject retains interest in emotional and physical intimacy or sexual activity
NSA 11	Poor grooming and hygiene	The subject presents with poorly groomed hair, dishevelled clothing, etc.
NSA 12	Reduced sense of purpose	This item assesses whether the subject possesses integrated goals for his/her life
NSA 13	Reduced interests	This item assesses the range and intensity of the subject’s interests
NSA 14	Reduced daily activity	This item assesses the level of the subject’s daily activity and his/her failure to take advantage of the opportunities his/her environment offers
NSA 15	Reduced expressive gestures	Gestures and body movements that normally facilitate communication during speech are less than normal, or are not observed at all
NSA 16	Slowed movements	This item assesses how much the subject’s voluntary movements are slowed. At a minimum, one should rate movements as gait and those of rising from a chair
NSA 17	Global negative symptoms rating	This item assesses the overall impression of negative symptoms in the subject
NSA 18	NSA total	Sum of the ratings from questions 1-16
NSA 19	NSA communication	Sum of the ratings from questions 1-4
NSA 20	NSA emotion affect	Sum of the ratings from questions 5-7
NSA 21	NSA social involvement	Sum of the ratings from questions 8-10
NSA 22	NSA motivation	Sum of the ratings from questions 11-14
NSA 23	NSA retardation	Sum of the ratings from questions 15-16

### 2.3 Acquisition of non-verbal speech features

#### 2.3.1 Equipment and procedure

The system deployed in this paper is based on our earlier work [37, 38], where we developed a machine learning based system that is able to infer the levels of interest, dominance, and agreement with 85%, 86% and 82% accuracy respectively from dyadic conversations. We employed easy-to-use portable equipment for recording conversations; it consisted of lapel microphones for each of the two speakers and an audio H4N recorder that allowed multiple microphones to be interfaced with a laptop. The audio data was recorded as a 2-channel audio.wav file (one channel for each speaker). This file allows us to detect which speaker is speaking at any given time.

The patient and the psychologist wore their respective microphones during the whole interview of NSA and the whole conversation was recorded. There was no pre-determined duration for the interview; instead it depended on participant’s response to the questions asked by the psychologist. On average, the interviews lasted about 30 minutes. Before the actual recording, we ensured that all the devices are connected. During the interview the software applications were monitored from another room via remote desktop to ensure the recording device functioned properly, and simultaneously maintain confidentiality of the participants’ speech.

#### 2.3.2 Extraction of non-verbal cues

**Fig 1:**
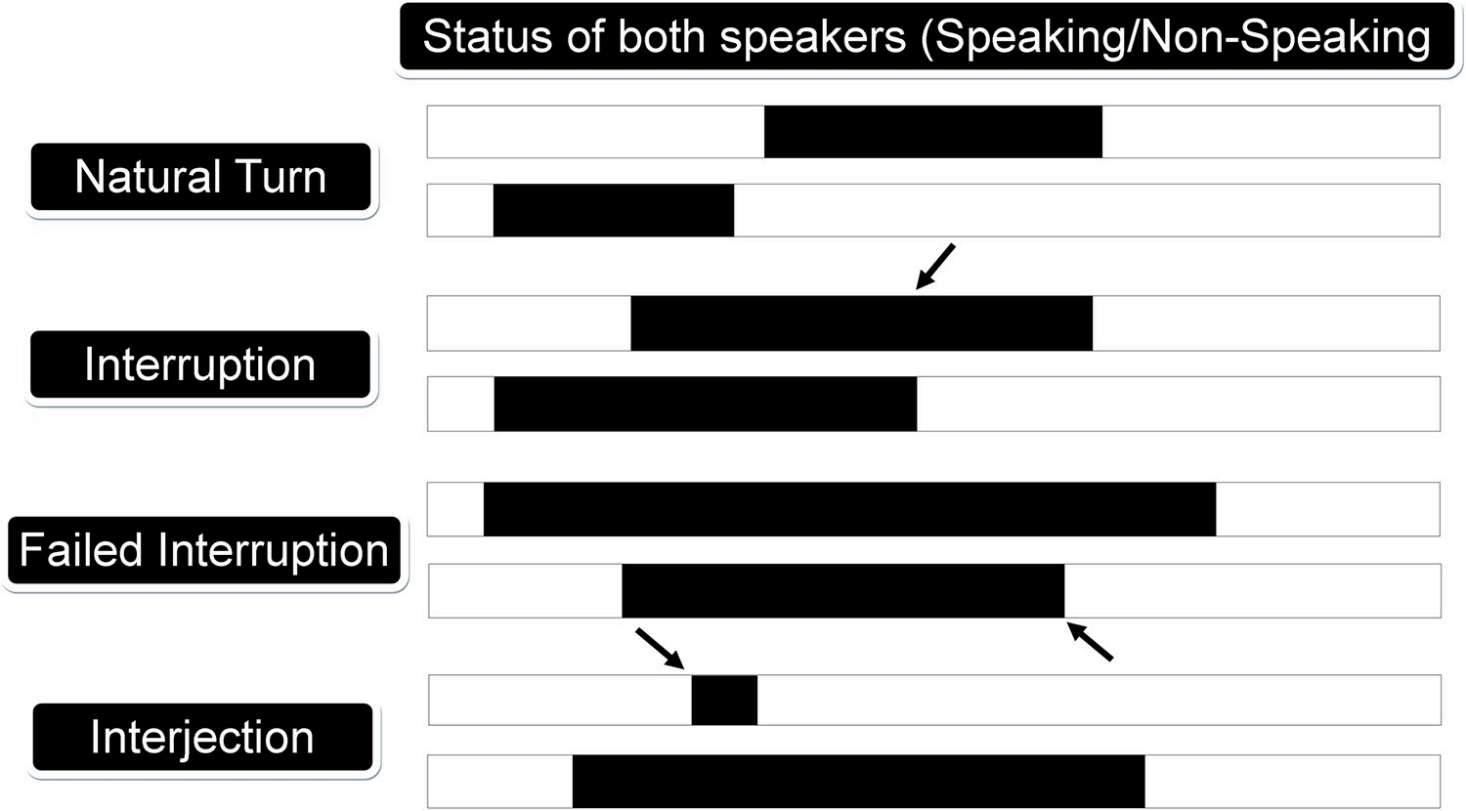
Illustration of conversational cues or features. Periods of speaking and non-speaking are indicated in black and white respectively.

We considered two types of low-level speech metrics: conversational and prosody related cues. The conversational cues accounted for who was speaking, when and by how much, while the prosodic cues quantified how people talked during their conversations. A detailed list of conversational cues is showed in Table 3. We used Matlab to compute the following conversational cues: the number of natural turns, speaking percentage, mutual silence percentage, turn duration, natural interjections, speaking interjections, interruptions, failed interruptions, speaking rate and response time [37]. Fig 1 explains extraction of some of the conversational cues.

**Table 3:** List of conversational, and prosodic features.

Category	Features
**Conversational**	
Speaking duration	Speaking %, Mutual silence, Difference in Speaking %, Overlap, Response time
Speaking turns	Natural turns, Turn duration
Interruption	Interruptions, Failed interruptions
Interjection	Interjection, Speaking interjection
**Prosodic**	
Frequencies	Larynx frequency (F0), Formant (F1, F2, F3)
MFCC	Mel-frequency cepstral coefficients
Amplitude	Mean volume, Max volume, Min volume, Entropy

We considered the following prosodic cues: amplitude, larynx frequency (F0), formants (F1, F2, F3), and mel-frequency cepstral coefficients (MFCCs). These cues were extracted from 30 ms segments at a fixed interval of 10 ms; they tended to fluctuate rapidly in time. Therefore, we computed various statistics of those cues over a time period of several seconds, including minimum, maximum, mean and entropy. The prosodic features are the standard audio features used in research, but the conversational features have been designed specifically for dyadic conversations. Table 4 provides the definition for these conversational features.

**Table 4:** Explanation of non-verbal conversational cues.

Non-Verbal Feature	Description
Natural Turn-Taking	The number of times person ‘A’ speaks in the conversation without interrupting person ‘B’ (see Fig 1). Normalized to per minute.
Turn Duration	The average duration of a speaker’s turn.
Speaking %	The percentage of time a person speaks in the conversation.
Speaking % Difference	The difference between the speaking percentages of both speakers.
Mutual Silence %	The percentage of time when both participants are silent.
Interruption	Person ‘A’ interrupts person ‘B’ while speaking, and takes over. Person ‘B’ stops speaking before person ‘A’ does (see Fig 1).
Speaking Interjection	Short utterances such as ‘okay’, ‘hmm’ etc. when other speaker is speaking (see Fig 1).
Speech Gap	The gap that a person takes between his/her consecutive turns.
Response Time	If person ‘A’ finishes speaking, then the time taken for person ‘B’ to start speaking is called response time.

## 3 Statistical analyses

First, Matlab was used to test the Pearson’s correlation between the objective audio features and the subjective negative symptoms ratings. In the second step, we analyzed the automated prediction of negative symptoms from audio features. We determined the prediction accuracy for some NSA-16 criteria. The rating scale ranges from 1-6, where a value of 1 and 2 would be coded as non-observable group and and a value between 3 and 6 would be coded as observable. We then used classification algorithms to determine the accuracy with which observable and non-observable classes can be differentiated. We performed leave-one-patient-out cross-validation to calculate the accuracy and AUC for these criteria. For feature selection we utilized CFSsubset attribute selection [39], and Correlation attribute selection [40]. CFSsubset attribute selection evaluates the worth of a subset of features by considering the individual predictive ability of each feature along with the degree of intercorrelation between the features. Subsets of features that are highly correlated with the class while having low intercorrelation are preferred. Correlation attribute selection [40] evaluates the worth of an attribute by measuring the Pearson correlation between it and the class label. At the end we present the results for the classification of the healthy controls and individuals with schizophrenia. The classification was computed by leave-one-person-out cross-validation, i.e., for each participant the classifier was tested on the instances of that participant and trained on all the remaining instances.

## 4 Results

### 4.1 The correlations between non-verbal speech features and NSA scores

**Fig 2:**
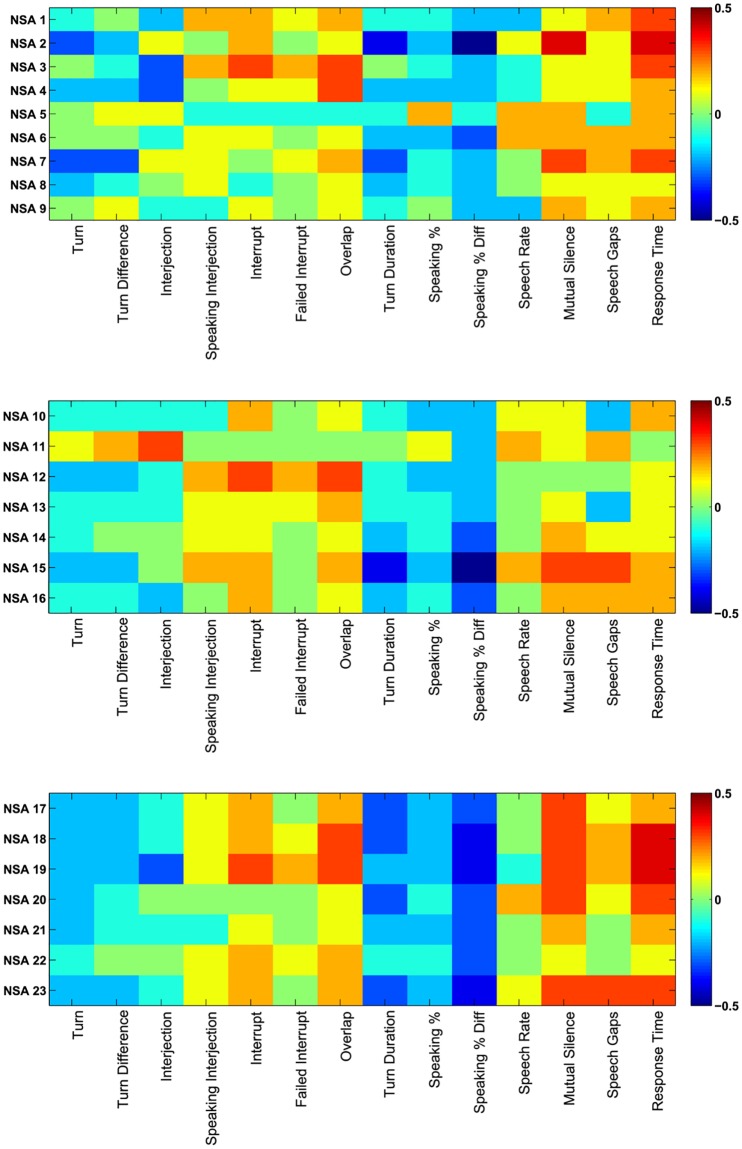
Colormap plots of NSA-16 ratings. Colormap plots between (a) NSA-16 features 1-9 and conversational features, (b) NSA-16 features 10-16 and conversational features, and (c) NSA-16 features 17-23 and conversational features.

The colormaps in Fig 2 showed the correlation of NSA-16 criteria with non-verbal conversational audio features, where Table 5 presents the values for significant correlations. It can be seen from the colormap that features like Failed Interrupt, Overlap, Mutual Silence, Speech Gap and Response Time are directly correlated to the negative symptoms; on the other hand Natural Turns, Interjections, Interrupts, Speaking Percentage and Turn Duration are inversely correlated to the negative symptoms.

**Table 5:** Correlation values between NSA and speech features.

	Natural Turn	Difference Turn	Interject	Speaking Interject	Interrupt	Failed Interrupt	Overlap	Speaking	Difference Speaking	Turn Duration	Speaking Rate	Mutual Silence	Speech Gap	Response Time
NSA 1	-0.074	-0.039	-0.229	0.195	0.241	0.091	0.169	-0.081	-0.076	-0.174	-0.155	0.117	0.245	0.307
	(0.593)	(0.782)	(0.096)	(0.158)	(0.079)	(0.515)	(0.223)	(0.563)	(0.584)	(0.209)	(0.265)	(0.399)	(0.075)	(0.024)
NSA 2	-0.276	-0.176	0.05	0.025	0.154	-0.035	0.098	-0.437	-0.178	-0.461	0.135	0.404	0.149	0.355
	(0.044)	(0.204)	(0.718)	(0.859)	(0.267)	(0.803)	(0.482)	(0.001)	(0.197)	(0.001)	(0.331)	(0.002)	(0.283)	(0.008)
NSA 3	-0.044	-0.055	-0.309	0.177	0.277	0.246	0.296	-0.027	-0.102	-0.227	-0.125	0.096	0.129	0.29
	(0.754)	(0.691)	(0.023)	(0.199)	(0.043)	(0.073)	(0.03)	(0.844)	(0.463)	(0.099)	(0.366)	(0.491)	(0.351)	(0.033)
NSA 4	-0.195	-0.205	-0.265	-0.016	0.088	0.137	0.255	-0.163	-0.24	-0.2	-0.146	0.119	0.054	0.216
	(0.158)	(0.137)	(0.053)	(0.908)	(0.527)	(0.324)	(0.063)	(0.238)	(0.081)	(0.147)	(0.293)	(0.39)	(0.697)	(0.116)
NSA 5	-0.005	0.132	0.138	-0.14	-0.112	-0.105	-0.121	-0.081	0.16	-0.061	0.185	0.172	-0.051	0.192
	(0.973)	(0.34)	(0.318)	(0.311)	(0.42)	(0.448)	(0.383)	(0.559)	(0.247)	(0.66)	(0.18)	(0.214)	(0.715)	(0.164)
NSA 6	-0.044	-0.048	-0.108	0.074	0.144	-0.012	0.126	-0.172	-0.165	-0.323	0.237	0.157	0.153	0.226
	(0.752)	(0.731)	(0.439)	(0.596)	(0.3)	(0.933)	(0.362)	(0.213)	(0.232)	(0.017)	(0.085)	(0.258)	(0.268)	(0.1)
NSA 7	-0.349	-0.326	0.053	0.102	0.019	0.144	0.248	-0.326	-0.14	-0.183	0.036	0.342	0.187	0.285
	(0.01)	(0.016)	(0.703)	(0.462)	(0.889)	(0.299)	(0.071)	(0.016)	(0.314)	(0.186)	(0.796)	(0.011)	(0.175)	(0.036)
NSA 8	-0.224	-0.076	-0.01	0.095	-0.056	-0.019	0.071	-0.224	-0.139	-0.162	-0.045	0.119	0.067	0.092
	(0.103)	(0.586)	(0.944)	(0.493)	(0.688)	(0.892)	(0.609)	(0.104)	(0.316)	(0.241)	(0.747)	(0.393)	(0.631)	(0.509)
NSA 9	-0.027	0.059	-0.073	-0.092	0.147	0.027	0.081	-0.07	0.014	-0.17	-0.158	0.219	0.137	0.226
	(0.846)	(0.671)	(0.599)	(0.508)	(0.29)	(0.846)	(0.559)	(0.613)	(0.918)	(0.219)	(0.255)	(0.112)	(0.323)	(0.1)
NSA 13	-0.068	-0.064	-0.105	0.062	0.091	0.072	0.171	-0.057	-0.105	-0.151	-0.019	0.054	-0.198	0.147
	(0.625)	(0.648)	(0.451)	(0.656)	(0.514)	(0.605)	(0.218)	(0.684)	(0.449)	(0.275)	(0.893)	(0.698)	(0.15)	(0.288)
NSA 14	-0.069	-0.023	0.027	0.111	0.103	0.029	0.14	-0.155	-0.101	-0.28	-0.026	0.17	0.12	0.084
	(0.618)	(0.87)	(0.848)	(0.426)	(0.459)	(0.834)	(0.314)	(0.263)	(0.465)	(0.04)	(0.853)	(0.22)	(0.387)	(0.548)
NSA 15	-0.222	-0.175	-0.047	0.188	0.165	0.035	0.172	-0.359	-0.232	-0.466	0.199	0.332	0.306	0.246
	(0.107)	(0.205)	(0.734)	(0.172)	(0.234)	(0.801)	(0.214)	(0.008)	(0.092)	(0.001)	(0.149)	(0.014)	(0.025)	(0.073)
NSA 16	-0.08	-0.125	-0.179	0.046	0.232	0.012	0.116	-0.161	-0.142	-0.273	-0.006	0.206	0.24	0.179
	(0.564)	(0.367)	(0.196)	(0.742)	(0.091)	(0.93)	(0.402)	(0.244)	(0.304)	(0.046)	(0.963)	(0.135)	(0.08)	(0.196)
NSA Global	-0.242	-0.168	-0.082	0.099	0.188	0.018	0.161	-0.273	-0.171	-0.311	0.02	0.271	0.118	0.161
	(0.077)	(0.225)	(0.557)	(0.476)	(0.173)	(0.898)	(0.244)	(0.046)	(0.215)	(0.022)	(0.886)	(0.047)	(0.396)	(0.246)
NSA 18	-0.24	-0.157	-0.131	0.109	0.248	0.105	0.263	-0.301	-0.217	-0.432	0.027	0.307	0.168	0.369
	(0.08)	(0.258)	(0.345)	(0.435)	(0.071)	(0.452)	(0.055)	(0.027)	(0.115)	(0.001)	(0.845)	(0.024)	(0.225)	(0.006)
NSA 19	-0.201	-0.157	-0.259	0.148	0.278	0.151	0.282	-0.248	-0.2	-0.377	-0.099	0.264	0.215	0.42
	(0.144)	(0.256)	(0.058)	(0.287)	(0.041)	(0.277)	(0.039)	(0.07)	(0.147)	(0.005)	(0.477)	(0.054)	(0.118)	(0.002)
NSA 20	-0.181	-0.112	0.03	0.021	0.032	0.012	0.122	-0.271	-0.076	-0.274	0.22	0.31	0.14	0.329
	(0.19)	(0.419)	(0.828)	(0.879)	(0.816)	(0.933)	(0.379)	(0.048)	(0.585)	(0.045)	(0.111)	(0.023)	(0.313)	(0.015)
NSA 21	-0.221	-0.112	-0.075	-0.051	0.145	0.03	0.12	-0.21	-0.173	-0.254	-0.043	0.188	-0.026	0.236
	(0.108)	(0.42)	(0.592)	(0.712)	(0.296)	(0.832)	(0.386)	(0.128)	(0.21)	(0.063)	(0.759)	(0.174)	(0.851)	(0.086)
NSA 22	-0.107	-0.036	0.002	0.131	0.197	0.118	0.239	-0.118	-0.129	-0.281	0.014	0.11	0.022	0.115
	(0.44)	(0.796)	(0.99)	(0.345)	(0.154)	(0.394)	(0.081)	(0.395)	(0.352)	(0.039)	(0.922)	(0.427)	(0.875)	(0.406)
NSA 23	-0.186	-0.178	-0.121	0.147	0.225	0.029	0.171	-0.317	-0.224	-0.443	0.127	0.321	0.321	0.251
	(0.179)	(0.198)	(0.385)	(0.29)	(0.103)	(0.834)	(0.216)	(0.02)	(0.104)	(0.001)	(0.359)	(0.018)	(0.018)	(0.067)

### 4.2 The automated prediction of negative symptoms from audio features

Table 6 presents the results for Prolonged time to respond, Restricted speech quantity, Impoverished speech content, Emotion: Reduced range, Affect: Reduced modulation of intensity, Reduced social drive, and Reduced expressive gestures along with the best five features in individuals with schizophrenia.

**Table 6:** Classification results for NSA criteria.

NSA Criteria	Algorithm	Feature Selection	Confusion Matrix		Accuracy	AUC	Best Features
			Non-Observable	Observable			
NSA 1	SVM	Correlation	33	4	79.6%	0.74	Ent_F2, Ent_F3, Ent_F1
			7	10			Speech_Gap, Response_Time
NSA 2	SVM	Correlation	26	7	70.4%	0.68	Max_Vol, Mean_Vol, Ent_Freq
			9	12			MFCC, Mutual_Silence
NSA 3	SVM	Correlation	18	9	59.3%	0.59	MFCC2, Overlap, Response_Time
			13	14			Failed_Interrupt, MFCC1
NSA 5	SVM	Correlation	13	12	53.7%	0.54	Ent_Vol, MFCC8, Max_Vol
			13	16			MFCC, Mean_Vol
NSA 6	SVM	Correlation	13	13	59.3%	0.59	Turn_Duration, Speech_Gap, MFCC5
			9	19			MFCC10, Response_Time
NSA 8	SVM	Correlation	6	7	74.1%	0.65	MFCC2, Speaking, MFCC4
			7	34			MFCC3, MFCC8
NSA 15	SVM	CFSsubset	30	5	77.8%	0.74	Turn_Duration, Ent_Freq, Mutual_Silence
			7	12			Max_Vol, Mean_Vol

### 4.3 Classification of patients v/s healthy subjects

Table 7 presents the patients vs healthy controls classification results for various machine learning algorithms along with the best five features. In the last row we present the statistics for a baseline classifier which classifies every participant as a patient. The highest accuracy is 81.3% which is really promising, because it shows that using non-verbal speech features we can differentiate between healthy individuals and individuals with schizophrenia.

**Table 7:** Patient vs healthy classification.

Algorithm	Feature Selection	Confusion Matrix		Accuracy	AUC	Precision (Patient)	Recall (Patient)	F-Score (Patient)	Best Features
		Patient	Healthy						
SVM	ReliefF	40	14	70%	0.68	0.8	0.74	0.77	speech_gap, ent_freq, mean_vol
		10	16						ent_f1, ent_f3
Random Forest	CFSsubset	42	12	72.5%	0.8	0.81	0.78	0.79	speaking, speech_gap, ent_freq
		10	16						ent_f3, mfcc
MLP	None	44	10	81.3%	0.9	0.9	0.82	0.85	
		5	21						
Ensemble (Bagging)	CFSsubset	46	8	77.5%	0.8	0.82	0.85	0.84	speaking, speech_gap, ent_freq
		10	16						ent_f3, mfcc

**Fig 3:**
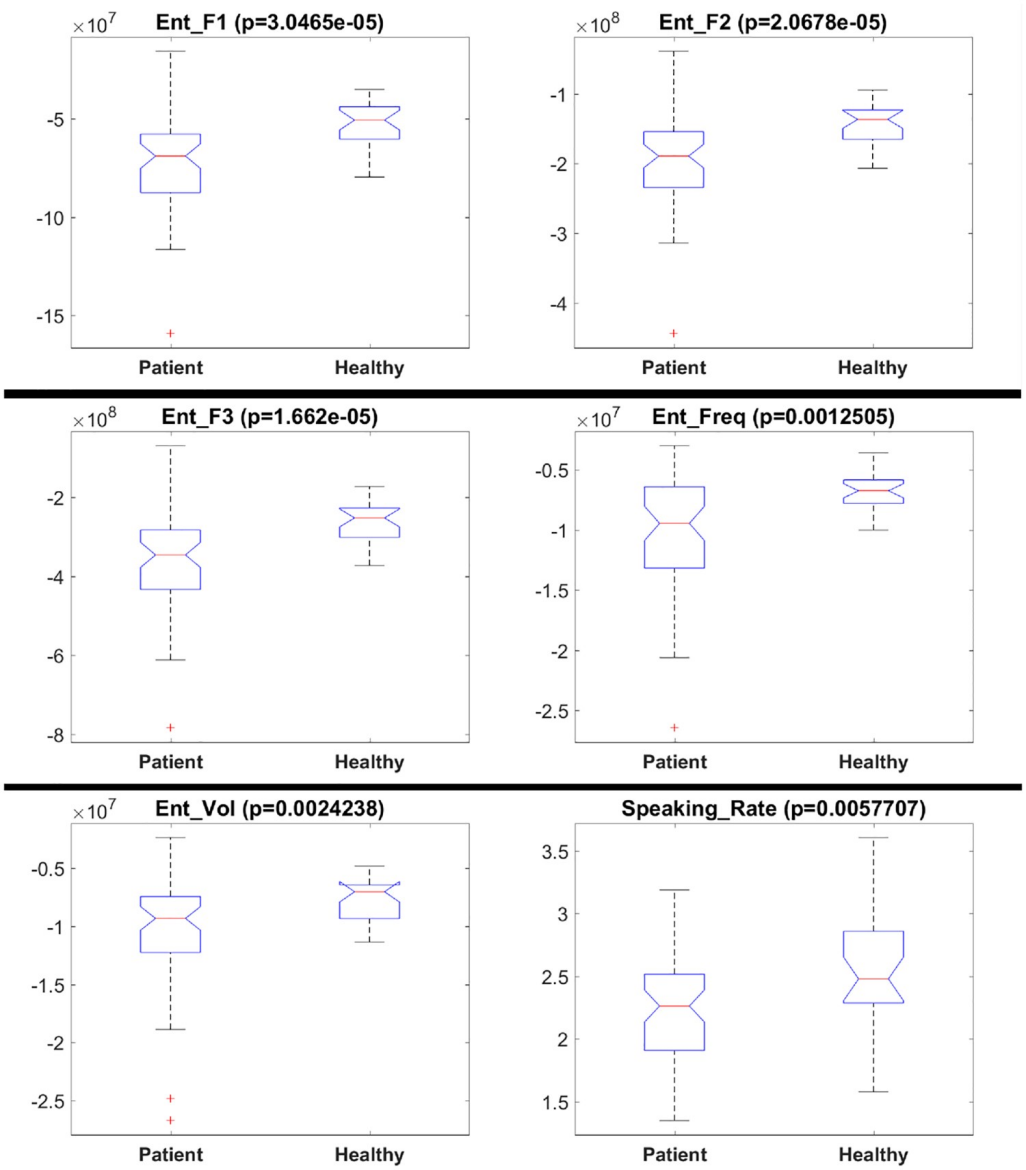
Boxplots for features that are significantly different for healthy subjects and patients. Boxplots for (a) F1 Entropy, (b) F2 Entropy, (c) F3 Entropy, (d) Frequency Entropy, (e) Volume Entropy, and (f) Speaking Rate.

Fig 3 presents boxplots and results (p-values) for the Kruskal-Wallis test. We tested whether a feature is significantly different for the healthy and patient groups. The plots are only shown for features with p-values below 0.01 for the Kruskal-Wallis test. It can be seen from the figures that frequency and volume entropies show significant difference among the prosodic features, while speaking rate shows significant difference among the conversational features. The healthy group has higher frequency and volume entropy as compared to the patient group. This finding implies that the healthy subjects speak in a less monotonous manner compared to the patients, and have more variability in the volume of their speech. Similarly, the speaking rate seems to be higher for the healthy group. These findings are in line with the results presented in [27], where speech rate significantly discriminated patients and healthy controls, and [31], where patients were found to be less fluent.

## 5 Discussion

As can be observed from Fig 2, significant correlations exist between the subjective ratings (NSA-16 items) and the objective measures (conversational features). An interesting point to note here is that the absolute values of the correlations are higher in Fig 2(a) and 2(c) compared to those in Fig 2(b). This difference can be attributed to the fact that NSA-16 items 1-9 (see Table 2) are closely associated with speech impairments. Consequently, these specific NSA-16 items are in greater congruence with the objective speech-related measures compared to the rest of the NSA-16 items, yielding higher absolute values of correlations. Similarly, Fig 2(c) contains the cumulative NSA-16 items, which is reflected in the overall higher absolute correlations.

Table 5 presents the correlation values for the NSA-16 items and speech features, providing the complete picture by listing all the correlations and their corresponding p-values. It can be noted that features such as Failed Interrupts, Response Time and Overlap correlate directly with the negative symptoms, i.e., cases which saw an increased Response Time or Overlap during the interview also saw a higher rating on equivalent items of the subjective NSA-16 scale, indicating greater severity of negative symptoms. The reverse situation occurred with the features such as Natural Turns, Speaking %, or Turn Duration; the reduced values of such features, associated often with disjoint communication, saw increased ratings on the speech-related NSA-16 items, resulting in negative correlations.

It can be seen from the Table 5 that Response Time has significant positive correlations with the NSA criteria Prolonged Time to Respond, Restricted Speech Quantity, Impoverished Speech Content, NSA Total, NSA Communication, and NSA Emotion Affect. Mutual Silence has significant positive correlations with the NSA criteria Restricted Speech Quantity, Affect: Reduced Display on Demand, NSA Total, NSA Communication, NSA Emotion Affect, and NSA Retardation. Speaking % has significant negative correlations with the NSA criteria Restricted Speech Quantity, Affect: Reduced Display on Demand, NSA Total, NSA Emotion Affect, and NSA Retardation. Turn Duration has significant negative correlations with the NSA criteria Restricted Speech Quantity, Affect: Reduced Modulation of Intensity, NSA Total, NSA Communication, NSA Emotion Affect, and NSA Retardation.

An interesting observation is the significant correlations between Reduced Expressive Gestures with non-verbal speech features. It has significant positive correlation with Mutual Silence, and Speech Gap, showing a decrease in gesture usage if there is more silence. On the other hand Reduced Expressive Gestures has significant negative correlation with Turn Duration, and Speaking %, which shows that the gesture usage increases with increase in speech.

The results in Table 6 present the detection accuracies for Prolonged time to respond, Restricted speech quantity, Impoverished speech content, Emotion: Reduced range, Affect: Reduced modulation of intensity, Reduced social drive, and Reduced expressive gestures. We achieve higher accuracy of 79.6%, 77.8% and 70.4% for Prolonged time to respond, Reduced expressive gestures and Restricted speech quantity. For Impoverished speech content, Emotion: Reduced range, and Affect: Reduced modulation of intensity, we achieve rather moderate accuracies of 59.3%, 53.7%, and 59.3%. The NSA item Reduced social drive has a large imbalance between non-observable and observable classes. These results correspond to Table 5, where high correlation values lead to a better prediction in most of the cases.

The results in Table 7 present the accuracies for patient v/s healthy classification. The highest accuracy of 81.3% was achieved by Multi-layer perceptron. Other algorithms like SGD, Random Forest, and Random-subspace (ensemble) achieve 70.0%, 72.5%, and 77.5% respectively [41].

In this paper we have presented our study on the objective and automated analysis of the speech deficiencies associated with schizophrenia. Our study is unique in several aspects. First of all, our dataset contains 80 subjects, including 56 patients and 24 healthy controls, which is a larger group than in most related studies. Moreover, the recordings are about 30 minutes long, and are substantially longer than recordings in similar studies typically lasting only a few minutes. Also we have not edited the recordings in any way, and analyzed the entire recordings, instead of selecting particular segments. As a result, our approach could be applied in realistic settings such as face-to-face interviews or phone calls, as there is no need for manual editing.

Moreover, the semi-structured nature of our participant interviews attempts to recreate an environment that is close to real-life clinical settings. We are interested in the social and cognitive behavioral patterns of the participants in their everyday lives, hence we decided not to apply any external stimuli, in contrast to earlier studies [29].

Our approach is more comprehensive compared to earlier studies, as we combine both prosodic as well conversational speech cues; this allowed us to conduct a more in-depth analysis. These objective cues, once validated through their strong correlations with established subjective measures, were utilised to predict the aforementioned subjective measures and to distinguish patients from healthy controls. The conversation speech cues used in this study and the correlations between NSA items and these conversational features are unique to our research.

A few earlier studies describe their attempt to develop automated methods to analyze audio and speech features of individuals suffering from schizophrenia. However, these approaches have the following limitations. Either the studies only consider prosodic cues related to flattening of affect or alogia as in [29], [30], [31], and [18], or are limited in the duration of speech data as in [30] (duration—1 minute), [31] (duration—10 minutes) and [32] (first paragraph read from a medieval classic). Only Cohen et al. [27] explored a single conversational feature (speech rate) together with other prosody features (inflection). None of the above studies utilized these speech features to distinguish between the patients and healthy controls with the exception of the one conducted by Martinez et al. [32], who achieved a classification accuracy of 93.8%. However, as pointed out earlier, their data is of rather short duration, and hence, these results may be less reliable compared to our results, obtained from 30 minutes recordings. Such long-term recordings give us the opportunity to explore the correlations between non-verbal speech cues and negative symptoms in individuals with schizophrenia with greater depth. We believe that more studies of this nature are required, with more and longer recordings in realistic settings, to fully establish the effectiveness of non-verbal audio cues for assessing the negative symptoms of schizophrenia. These results can be the stepping stone towards building an automated tool which could predict negative symptoms by analyzing the speech of a patient in an automated manner. The results of our patient v/s healthy classification analysis are also very promising. It shows that participants can indeed be classified as individuals with schizophrenia or as healthy individuals on the basis of their non-verbal speech features.

Also, in the future, we plan to explore the temporal variation of the speech cues for the subjects and controls, specifically, how their speech features change over sessions, and, if at all, in different manner for subjects and controls. In this paper, we have only investigated the non-verbal cues related to speech. Visual non-verbal cues such as posture, gestures, etc., have not been investigated here, which we plan to address in the future.

The present study is not without limitations. As this is a cross-sectional study, no conclusion could be drawn about the stability of the relationship between the objective measure and subject measure over time. Some factors that might affect negative symptoms and speech production such as participants’ smoking history and extrapyramidal symptoms were not assessed and controlled in this study. The correlations between the objective speech cues and NSA ratings ranged between 0.5 and -0.5. Therefore there were a big percentage of variance could not be explained. In addition, this study only tested the relationship between objective measures and subjective measures of negative symptoms in schizophrenia. Future study might want to examine this relationship in other psychiatric disorders such as depression or bipolar disorder to explore whether the relationship is generalizable to other psychiatric conditions.

## 6 Conclusion

In this paper we presented our findings regarding the correlations between the non-verbal speech cues and negative symptom ratings. Existing schizophrenia related research mostly focuses on semantic analysis and natural language processing. Little attention has been given to non-verbal speech features. In this paper we have highlighted the significance of these non-verbal speech features by showing their correlations with the negative symptoms for schizophrenia.

The results of our analysis are promising as there are significant correlations between non-verbal speech features and NSA-16 ratings. We also predicted NSA-16 criteria using machine learning algorithms trained on subjective ratings. The results show that some of the NSA-16 criteria can be classified as observable and non-observable using non-verbal speech features with quite high accuracy.

The positive findings from our analysis pave the way for identifying speech characteristics as markers for negative symptoms, developing a technological application that detects clinically significant speech patterns may assist clinicians in postulating functional level of an individual. With the gathered data from this study, we will work towards the development of a prototype, possibly as a mobile application to facilitate implementation.
